# Checklist of Fishes at Pergau Lake, Jeli, Kelantan, Malaysia

**DOI:** 10.21315/tlsr2019.30.1.10

**Published:** 2019-01-31

**Authors:** Malisa Ilyana Mohd Alias, Kamarul Hambali, Aainaa Amir, Norashikin Fauzi, Hizami Hassin, Sow Ai Yin

**Affiliations:** 1 Faculty of Earth Science, Universiti Malaysia Kelantan, 17600 Jeli, Kelantan, Malaysia; 2 Department of Biology, Faculty of Science, Universiti Putra Malaysia, 43400 UPM Serdang, Selangor, Malaysia

**Keywords:** Fish Diversity, Freshwater Fishes, Pergau Lake, Jeli, Kepelbagaian Ikan, Ikan Air Tawar, Tasik Pergau, Jeli

## Abstract

A survey was conducted to determine the diversity of fish in Pergau Lake, Jeli, Kelantan, Malaysia from 2 September to 18 October of 2016. Fish samples were collected at seven random sampling stations around the lake by using setting trap method. The collected samples were preserved in 10% formalin solution and delivered to the laboratory for further identification process. Fourteen species, namely as *Hemibagrus nemurus*, *Hampala macrolepidota*, *Clarias batrachus*, *Channa striata*, *Cyprinus carpio*, *Poropuntius smedleyi*, *Pangasius* sp., *Oreochromis niloticus*, *Oreochromis mossambicus*, *Leptobarbus hoevenii*, *Neolissochilus hexagonolepis*, *Tor tambroides*, *Osteochilus hasselti* and *Neolissochilus soroides*, comprising of six families were collected during this study. From this study, family of Cyprinidae was the dominant species in Pergau Lake with exactly 50% of catch percentage, followed by family of Bagridae (29%), Cichlidae (10%), Clariidae (5%), Channidae (3%) and Pangasiidae (3%). This study shows that there is still a diversity of fish species in Pergau Lake, showing that the water and its environmental condition is very good.

## Abstract (ms)

Satu tinjauan telah dijalankan untuk menentukan kepelbagaian ikan di Tasik Pergau, Jeli, Kelantan, Malaysia pada 2 September hingga 18 Oktober 2016. Sampel ikan telah dikumpulkan secara rawak di tujuh stesen pensampelan sekitar tasik menggunakan kaedah perangkap. Sampel yang dikumpul diawet di dalam 10% larutan formalin dan dihantar ke makmal untuk proses pengecaman. Empat belas spesis, *Hemibagrus nemurus*, *Hampala macrolepidota*, *Clarias batrachus*, *Channa striata*, *Cyprinus carpio*, *Poropuntius smedleyi*, *Pangasius* sp., *Oreochromis niloticus*, *Oreochromis mossambicus*, *Leptobarbus hoevenii*, *Neolissochilus hexagonolepis*, *Tor tambroides*, *Osteochilus hasselti* dan *Neolissochilus soroides*, terdiri daripada enam famili telah dikumpul sepanjang kajian ini dijalankan. Berdasarkan kajian, famili Cyprinidae adalah spesies yang dominan di Tasik Pergau, iaitu 50% daripada peratusan tangkapan, diikuti dengan famili Bagridae (29%), Cichilidae (10%), Clariidae (5%), Channidae (3%) dan Pangasiidae (3%). Kajian ini menunjukkan bahawa masih terdapat kepelbagaian spesies ikan di Tasik Pergau, yang menunjukkan keadaan air dan persekitaran adalah sangat baik.

## INTRODUCTION

Malaysia has been well known to be among the mega-diverse countries in terms of flora and fauna, and possesses various ecosystems and diversity for various life forms, including the fish fauna (Ambak *et al*. 2012). The water bodies in earth have covered many of different species and diversity of freshwater fishes (Ambak *et al*. 2012). There are five major fish habitats which are freshwater fish, marine fish, tropical fish, cold water and aquarium fish (Samat 2010). Each type of fish is determined by the habitats and characteristics of fish species which can indicate the variety of fish available in the world (Nelson 2006). The difference between freshwater fish and other types of fish is because they found in the water bodies such as lakes and rivers in which the salinity is less than 0.05% (Gene e*t al*. 2009).

There are many lakes and reservoirs in Malaysia that are mostly are man-made (Mohsin & Ambak 1996). Pergau Lake is a man-made lake which is located 100 kilometres from the Jeli city which is famous for its own beauty among nature lovers. It encompasses a 460-hectare of lake which flows with seven other rivers, producing high productivity and rich diversity of fish population. These river and water bodies play important roles in maintaining the number of fish population in the reservoir. Since the construction of hydroelectric dam near the Pergau area, the population of fish has changed and decreased. Pergau Lake is also well known as an eco-tourism destination whereby many natural activities can be done. There is also a small jetty built to allow tourist to enjoy the scenery via a boat ride. These activities are affecting the abundance of fish and water quality. Therefore, this study was carried out to analyse the presence of fish diversity in this lake.

## MATERIALS AND METHODS

**Figure 1:**
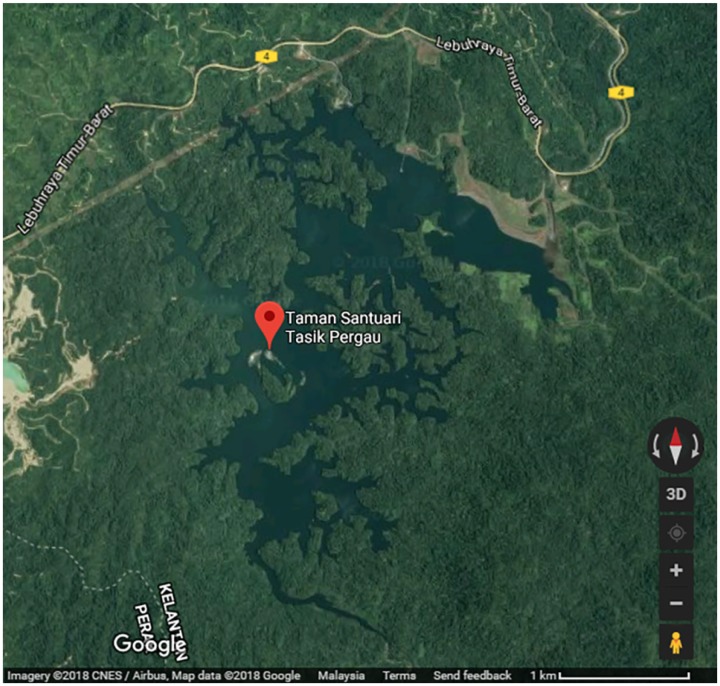
Study area.

Seven sampling stations around Pergau Lake were chosen randomly for this study area (Fig. 1). Setting traps with model 0.15 mm, mesh 6 cm, depth 30 md and length A (30) were used by two operators along the study period started from 2 September to 18 October 2016. This study was conducted three days a week during the study period. The traps were deployed at each station at 1830 hours and collected in the next day morning. The time of traps deployment and retrieval was recorded. The fish samples were collected and preserved in 10% formalin solution and delivered to the laboratory for further identification process. The fishes were identified up to the species level by using standard taxonomic keys proposed by Ambak *et al*. (2012). Each specimen was then measured for its total length and weight. This study also used the Simpson’s Diversity Index to measure species diversity. In ecology, it often used to quantify the biodiversity of a habitat. It takes into account the number of species present, as well as the abundance of each species.

## RESULTS AND DISCUSSION

A total of 14 species comprising of six families were recorded during the present study period (Table 1). The fish species recorded in the present study is higher than EIA (1987) study which recorded only 11 species. However, the total fish species in the present is lower when compared with the previous study by Amir *et al*. (2017) which recorded 17 species. *Hemibagrus nemurus* is the most dominant fish species caught in most of the stations followed by *Hampala macrolepidota*. However, from the interview with the anglers and South Kelantan Development Authority (KESEDAR) officer, *Channa micropeltes* has the most dominant species among all of the fishes. This might occur due to the use of bigger fish trap and fishing rod as their tools as the *Toman* fish is commonly in bigger size than other fish species in Pergau Lake. This study found that the size range and average size for each species are different. The tools used to measure the size and length of fish sample was the measuring tape. The biggest size range of fish sample was up to 72 cm that was *Leptobarbus hoevenii*. The smallest fish can be found at

Pergau Lake was *Tor tambroides* which ranged from 14.4 cm to 27.2 cm. The other fish specimens that were caught are namely *Clarias batrachus*, *Channa striata*, *Cyprinus carpio*, *Poropuntius smedleyi*, *Pangasius* sp., *Oreochromis niloticus*, *Oreochromis mossambicus*, *Leptobarbus hoevenii*, *Neolissochilus hexagonolepis*, *Tor tambroides*, *Osteochilus hasselti* and *Neolissochilus soroides*. Pergau Lake was the place for fish spawning dominated by *Oreochromis niloticus* and *Oreochromis mossambicus* which is managed by KESEDAR and the Rubber Industry Smallholders Development Authority (RISDA). They put a lot of fish cages which are 26 all together for KESEDAR and a few cages for RISDA. However, these cages were destroyed due to the decrease of water level from the dam’s construction. The fish died due to the low level of oxygen that is not suitable for *Oreochromis niloticus* and *Oreochromis mossambicus* to live. These species started to decrease and swim away to find new habitat and breeding ground. The shift in the dominant fish species caught commercially is a clear indication that the dynamics of the fish populations and their composition has changed over time (Lee *et al*. 2013).

**Figure 2:**
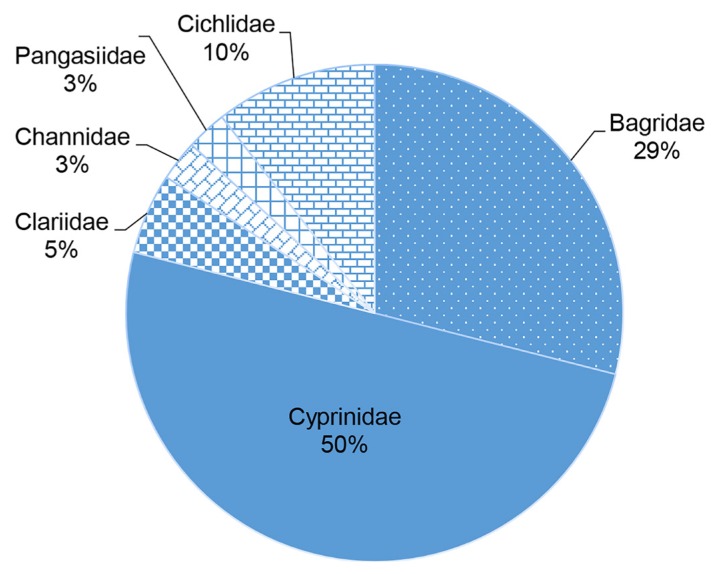
Percentage of fish species by family captured based on date collected at seven random sampling stations around Pergau Lake, Jeli, Kelantan.

This study found that Cyprinidae is the dominant fish family in Pergau Lake with exactly 50% of catch. This study has a few similarities with most studies conducted in freshwater areas in Malaysia which indicate that family Cyprinidae recorded the highest percentage of catch in freshwater habitats (Lee *et al*. 2013; Ismail *et al*. 2013; Nurul *et al*. 2016; Farinordin *et al*. 2016). According to Ambak *et al*. (2012), Cyprinidae is the largest family of freshwater fishes. In Malaysia, it is considered as the largest freshwater fish family in terms of number of genera and species and its abundance in rivers and lakes. Besides that, most Cyprinidae are riverine and spawned during the first rainy season after a long period of drought as they exhibit a variety of spawning habits (Ambak *et al*. 2012). The second dominant family is Bagridae which is also dominant in all station with 29% of population. The least number of families are Channidae and Pangasiidae. They have the same percentage of catch which are 3% (Fig. 2).

Based on this study, Pergau Lake has high diversity of fish (Simpson’s Diversity Index; D = 0.83). As mention in Simpson (1949), one area will show high diversity of species when the value of Simpson’s Diversity Index (D) is approaching to 1. This shows that the water quality is very good along with its environmental condition. However, control or monitoring of non-native species should be conducted by the authorities to prevent the extinction of the native species. Besides, further study should be carried out and it is recommended to use variety of sampling gears such as gillnets, fish traps, fishing rods and cast nets to gain more information on fish biodiversity and population in the Pergau Lake.

## CONCLUSION

This study shows that Pergau Lake still has high diversity of fish species. This indicate that the water quality and the environment of this lake are in a good condition. The authorities should monitor and maintain the Pergau Lake to ensure the sustainability of this area. Furthermore, depth studies are need to be conducted in order to obtain more information on fish diversity in Pergau Lake.

## Figures and Tables

**Table 1:** Fish species that has been sampled and recorded during the present study compared with previous study.

Family	Species	Local name	EIA-UKM (1987)	Amir *et al*. (2017)	Present study
Bagridae	*Hemibagrus nemurus*	Baung	−	+	+
Channidae	*Channa micropeltis*	Toman	−	+	+^*^
	*Channa gachua*	Haruan bukit/pecat	−	+	−
	*Channa striata*	Haruan	−	−	+
Cichlidae	*Oreochromis niloticus*	Tilapia merah	−	+	+
	*Oreochromis mossambicus*	Tilapia hitam	−	−	+
Clariidae	*Clarias batrachus*	Keli kayu	−	−	+
Cyprinidae	*Poropuntius smedleyi*	Tengas daun	+	+	+
	*Neolissochilus hexagonolepis*	Tengas	+	+	+
	*Neolissochilus soroides*	Kelah putih	−	−	+
	*Epalzcorhyncous siamensis*	Selimang siam	+	−	−
	*Puntius binotatus*	Tebal sisik	+	−	−
	*Osteochilus spilurus*	Rong	+	−	−
	*Osteochilus hasselti*	Terbol	−	−	+
	*Mystacoleucus marginatus*	Sia	+	+	−
	*Mystacoleucus chilopterus*	Sia	+	−	−
	*Hampala macrolepidota*	Sebarau	−	+	+
	*Cyclocheilichthys apogon*	Temperas	−	+	−
	*Barbonymus schwanenfeldii*	Lampam sungai	−	+	−
	*Cyprinus carpio*	Lee koh	−	+	+
	*Leptobarbus hoevenii*	Jelawat	−	+	+
	*Tor tambroides*	Kelah	−	+	+
Eleotridae	*Oxyeleotris marmoratus*	Ketutu	−	+	−
Nemacheilidae	*Nemacheilus* sp.	Pasir	+	−	−
Notopteridae	*Notopterus notopterus*	Selat	−	+	−
Pangasiidae	*Pangasius* sp.	Patin	−	+	+
Pristolepididae	*Pristolepis fasciatus*	Patong	−	+	−
Siluridae	*Silurichthys hasseltii*	Anak tapah	+	−	−
Sisoridae	*Glyptothorax major*	Depu	+	−	−
	*Glyptothorax platypogonoides*	Kenerak batu	+	−	−

*Note*: + = present; − = absent; +^*^ = present based on the interview with local anglers.
